# Supplementary material for: Decellularized rat brain extracellular matrix effectively induces the dopaminergic differentiation of human adipose-derived stem cells
Source: PLoS One. 2025 Sep 2;20(9):e0320367. doi: 10.1371/journal.pone.0320367 (PMC12404474; doi:10.1371/journal.pone.0320367)
Supplement: S1 Data — (PDF) [file pone.0320367.s001.pdf]

S1 Data. Raw images of RT-PCR analysis, related to Fig 3 and Fig 5.

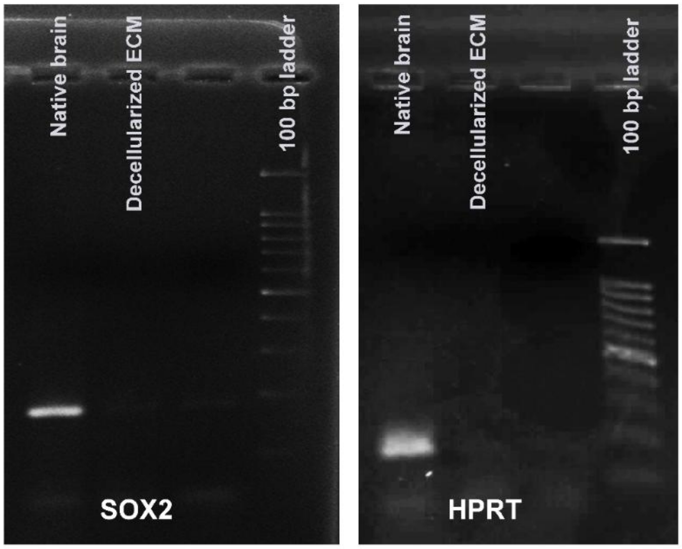

RT-PCR analysis for the expression of *SOX2* and *HPRT* in native and decellularized brain.

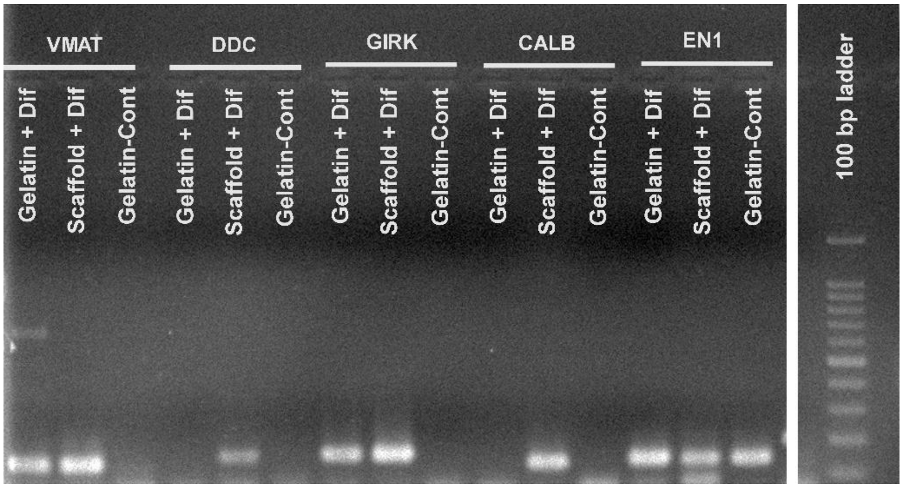

RT-PCR analysis for the expression of *VMAT, DDC, GIRK1, CALB,* and *EN1* genes.

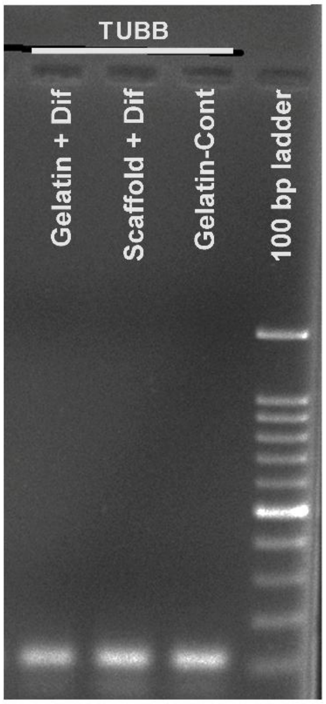

RT-PCR analysis for the expression of *TUBB* gene.

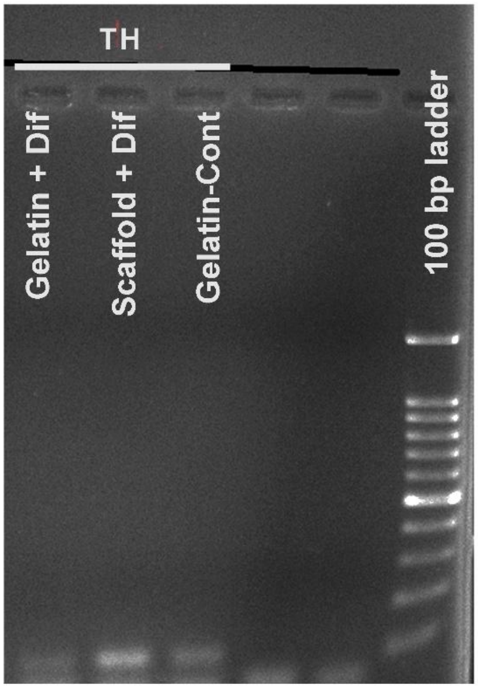

RT-PCR analysis for the expression of *TH* gene.

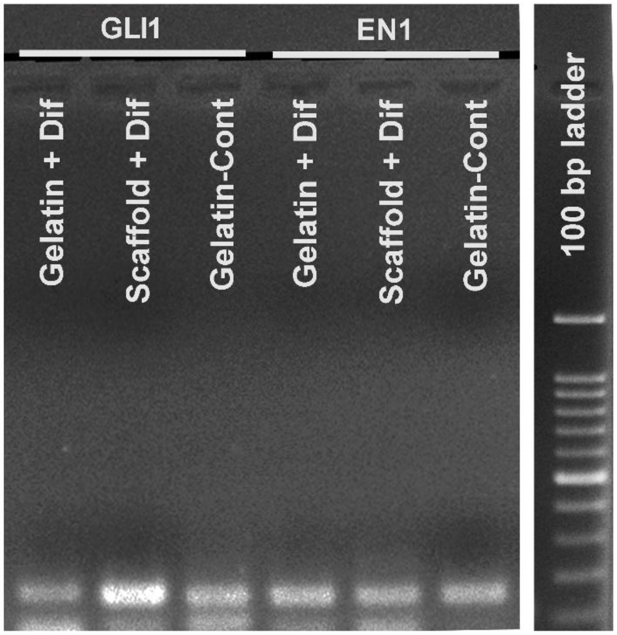

RT-PCR analysis for the expression of *GLI1 and EN1* genes.

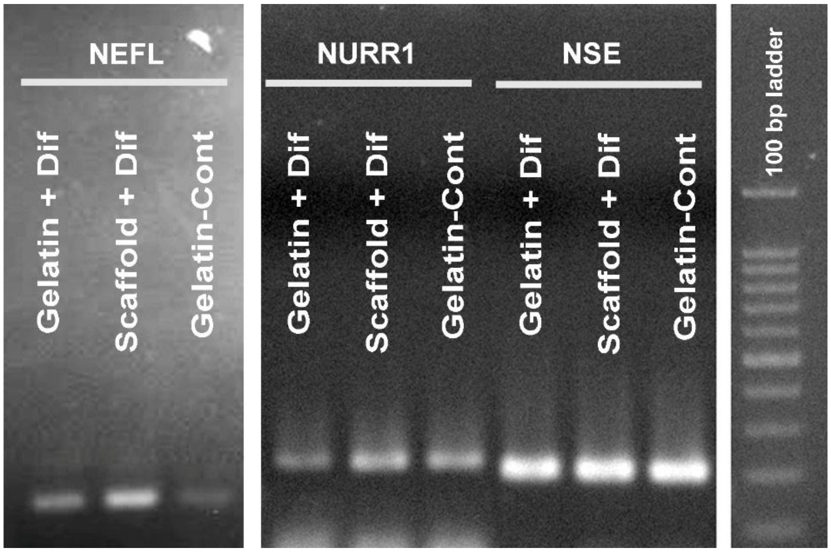

RT-PCR analysis for the expression of *NEFL, NURR1 and NSE* genes.
