# Supplementary material for: Decellularized rat brain extracellular matrix effectively induces the dopaminergic differentiation of human adipose-derived stem cells
Source: PLoS One. 2025 Sep 2;20(9):e0320367. doi: 10.1371/journal.pone.0320367 (PMC12404474; doi:10.1371/journal.pone.0320367)
Supplement: S2 Data — (PDF) [file pone.0320367.s002.pdf]

S2 Data. Raw images of western blots, related to Fig 7.

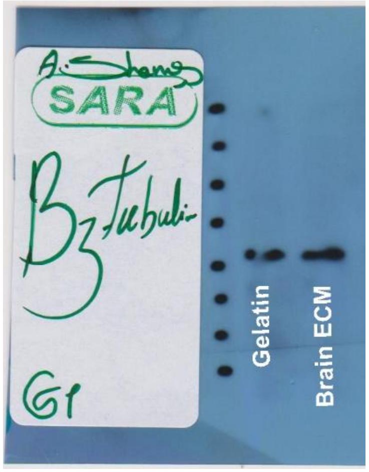

Western blot analysis for the expression of TUJ1 protein.

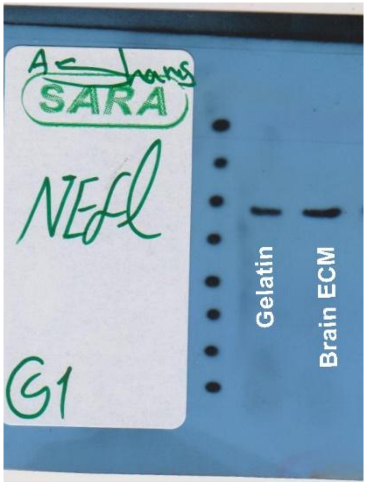

Western blot analysis for the expression of NEFL protein.

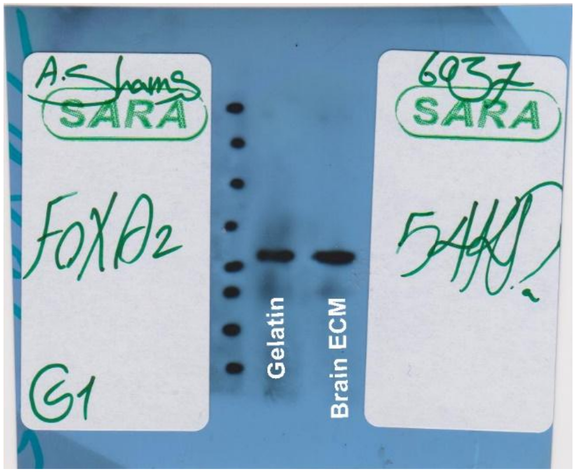

Western blot analysis for the expression of FOXA2 protein.

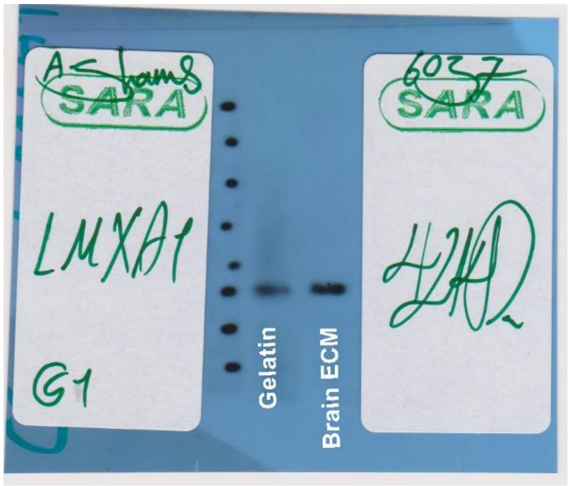

Western blot analysis for the expression of LMXA1 protein.

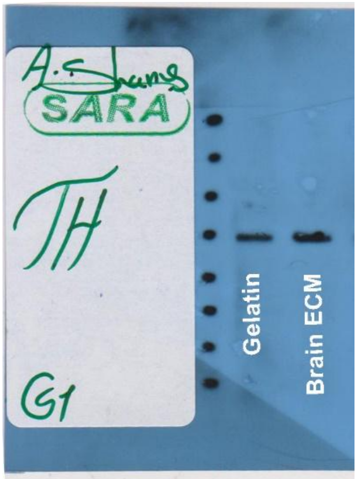

Western blot analysis for the expression of TH protein.

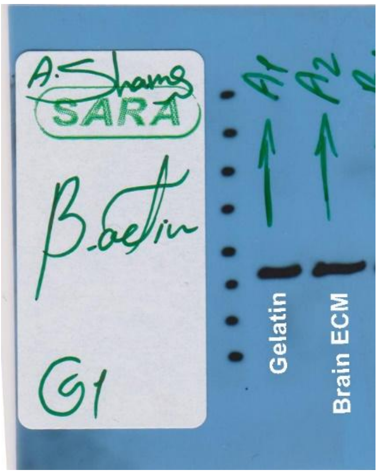

Western blot analysis for the expression of β-actin protein.
