# Supplementary material for: Decellularized rat brain extracellular matrix effectively induces the dopaminergic differentiation of human adipose-derived stem cells
Source: PLoS One. 2025 Sep 2;20(9):e0320367. doi: 10.1371/journal.pone.0320367 (PMC12404474; doi:10.1371/journal.pone.0320367)
Supplement: Attachment — Submitted filename: Review plos ONE dECM Brain ADPSCs.pdf [file pone.0320367.s005.pdf]

Decellularized rat brain extracellular matrix effectively induces the dopaminergic differentiation of human adipose-derived stem cells

Recommendation: *Minor Revision*. The manuscript generally meets PLOS ONE's publication criteria in terms of scientific rigor, completeness of data, and ethical standards. The study is well-designed and yields interesting results about using a decellularized rat brain extracellular matrix (ECM) to enhance dopaminergic differentiation of human adipose-derived stem cells (hADSCs). However, a few minor revisions are needed to improve clarity, consistency, and completeness. Below, I provide detailed comments on specific aspects:

Scientific Validity and Methodological Rigor

The experimental design is solid and appropriate for the research question. The authors compare hADSC differentiation on 3D decellularized brain scaffolds versus a standard 2D culture, which is a clear and logical way to test the influence of the ECM microenvironment. Strengths of the methodology include a thorough decellularization protocol (with chemical, enzymatic, and physical steps) and comprehensive validation of decellularization success (DAPI nuclear staining, H&E and Masson's trichrome histology, DNA quantification, laminin immunostaining, and SEM imaging). These confirm that cellular material was effectively removed while preserving ECM structure – lending credibility to the scaffold's integrity. The hADSCs are well-characterized prior to use (positive for MSC markers CD105, CD73, CD90 and able to undergo adipogenic/osteogenic differentiation), ensuring the starting cell population is appropriate and multipotent. The differentiation protocol is detailed and uses known dopaminergic-inductive factors (Neurobasal medium with SHH, FGF8, bFGF, later adding BDNF), which is scientifically sound. Multiple outcome measures are employed – including RT-PCR/qPCR for gene expression, immunocytochemistry/immunohistochemistry for neuronal markers, and Western blotting for protein levels – providing convergent evidence to support the conclusions. Overall, the study's methods and analyses are rigorous and support the validity of the findings.

Areas for improvement: A few clarifications could further strengthen the rigor.

1. the authors should quantify some of the qualitative observations. For example, immunostaining revealed a "significant proportion" of cells positive for TH and TUJ1 in

both conditions – it would improve the paper to quantify the percentage of cells expressing these markers on the scaffold vs. on 2D culture. This would give the reader a clearer idea of differentiation efficiency and how much the 3D ECM improves outcomes (e.g. "X% of cells were TH-positive on ECM vs. Y% on 2D"). If such quantification was done, it should be reported; if not, even a rough estimate or qualitative statement in the **Discussion** noting the difference in positive cell abundance would be helpful.

2. While the upregulation of dopaminergic genes (e.g. **TH, GLI1, VMAT2, GIRK2**) and exclusive expression of **DDC and CALB** in the 3D condition strongly support the conclusion, the manuscript could discuss the **functional maturity** of these differentiated cells. The authors are not expected to perform new experiments, but a brief acknowledgment that *functional* confirmation (such as dopamine release assays or electrophysiological properties) was beyond this study's scope would give a balanced perspective. This could be framed as a future direction, reinforcing that the current evidence of marker expression is sufficient to call them "dopaminergic-like" cells, while true functional neurons would require additional verification.
3. the inclusion of a control group where hADSCs are cultured on the scaffold **without** dopaminergic factors (to test if the ECM alone induces any neural differentiation) was not described. If such a control was not done, consider mentioning it as a potential future experiment to isolate the scaffold's inherent inductive effect.

These are minor suggestions to discuss or clarify; they do not detract from the core findings, which remain scientifically sound.

Reproducibility and Data Transparency

The manuscript provides **enough methodological detail for reproducibility**. The decellularization procedure is outlined step-by-step with concentrations, durations, and equipment, which would allow other researchers to replicate the scaffold preparation. The source and preparation of hADSCs and the differentiation protocol are also described with sufficient detail (including media composition and growth factor concentrations). The use of multiple biological sources — **five donors** for adipose tissue and **five rats** for brain scaffolds — is commendable, as it increases confidence that the results are not from a single sample bias. It would be helpful to

explicitly state how many independent experiments or biological replicates were analyzed for key results (e.g. were cells from all 5 donors used in parallel differentiations on separate scaffolds? or were some analyses done in triplicate per condition?). Clarifying the **number of biological replicates** for the gene expression and protein analysis will underline the robustness of the data. Overall, the data presented (PCR gels, qPCR relative expression, immunostaining images, and Western blots) appear comprehensive and support the conclusions drawn.

Areas for improvement: In line with PLOS ONE's data transparency policies,

1. The authors should ensure that all underlying data are made available. If not already done, include a Data Availability Statement indicating where numerical data **(e.g. the quantitative values behind the qPCR bar graphs or Western blot densitometry)** can be accessed. Additionally, when reporting statistical results, it's good practice to indicate the ***n*** and what it represents. For instance, if "four replicates" were used in qPCR, clarify if these are four technical replicates or four independent differentiations (possibly from multiple donors).
2. Minor statistical considerations: since multiple genes were compared between two conditions, the authors might note whether **any correction for multiple comparisons was applied or if each gene was treated as a separate planned comparison (the use of *t*-tests with Welch's correction is noted, which is appropriate for two-group comparisons assuming independent tests per gene/protein)**. These clarifications will further demonstrate the study's reproducibility and adherence to transparency standards.

Ethical Compliance and Documentation

The manuscript demonstrates **strong ethical compliance**. The authors clearly describe obtaining human adipose tissue with informed consent and approval, as well as the ethical oversight for animal use. Specifically, adipose samples were taken from patients undergoing elective surgery, with **verbal informed consent** documented by medical staff; this procedure was reviewed and approved by the institutional Bioethics Committee (approval ID provided: IR.NIGEB.EC.1400.2.26.C, dated 2021-05-16). The study states it was conducted in accordance with the Declaration of Helsinki, indicating attention to ethical standards for human tissue use. On

the animal side, five Wistar rats were used for brain tissue, with housing and care as per standard conditions and all surgeries performed under deep anesthesia to minimize suffering. The animal protocol was approved by the same Bioethics Committee (approval ID: IR.NIGEB.EC.1400.2.26.B). The inclusion of approval numbers and dates for both human and animal procedures, as well as mention of adherence to relevant guidelines (Helsinki declaration for humans, laboratory animal care guidelines for animals), is excellent and meets journal requirements.

Areas for improvement*:* Only minor clarifications are needed.

1. It would be helpful to **state the sex of the rats** used, if known, as reporting sex is encouraged for animal studies (e.g., "five male Wistar rats" or "five female Wistar rats"). Currently only the strain and weight are given.

Clarity of Writing and Presentation

In general, the manuscript is well-written and structured. The Introduction provides a clear rationale, explaining the importance of ECM in neural development and the gap in knowledge regarding brain ECM's effect on dopaminergic differentiation. The reader is given sufficient background to understand why this study matters (with citations to recent decellularization advances and prior uses of tissue-specific ECM). The Materials and Methods are detailed and logically ordered, and the Results flow in a coherent sequence (from stem cell characterization, to scaffold preparation/validation, to differentiation outcomes). The figures and tables cover all key data; for example, Figure 1 characterizes the ADSCs, Figures 2–4 likely show scaffold validation, Figure 5 covers gene expression, Figure 6 immunostaining, and Figure 7 Western blot results. This organization is easy to follow.

Areas for improvement: A careful proofread is needed to correct some minor typographical and grammatical errors. For instance, in the Figure 6 description, "againest" should be corrected to "against." In the Western blot methods, "ACBT served as the internal reference" appears to be a typo and should likely read "ACTB (β-actin) served as the internal reference protein." Consistency in terminology should be checked: the text sometimes refers to "LMXA1" (in the Results and figure labels) whereas the correct gene/protein name is LMX1A – using one consistent spelling

will avoid confusion. Similarly, “VMAT” is used in the text while the full name “VMAT2” is listed in the primers table; since VMAT2 is the specific vesicular monoamine transporter isoform measured, it may be clearer to use “VMAT2” throughout (or state at first use that “VMAT2 is referred to as VMAT”).

Overall, these are minor edits that will enhance clarity. The authors should also ensure all acronyms are defined at first mention (most are, such as ECM, hADSC, etc., but double-check in case any slipped through). The figure captions could be slightly expanded for self-contained clarity – for example, when mentioning “negative control” in immunostaining images, specify that it refers to omission of primary antibody.

Finally, one suggestion for the Discussion: consider explicitly stating the main take-home message in a clear concluding sentence. The Conclusion section does summarize the findings and their significance for future applications, which is good. With these small revisions and proofreading corrections, the writing will be of high quality and easy for readers to understand.
