# Supplementary material for: Decellularized rat brain extracellular matrix effectively induces the dopaminergic differentiation of human adipose-derived stem cells
Source: PLoS One. 2025 Sep 2;20(9):e0320367. doi: 10.1371/journal.pone.0320367 (PMC12404474; doi:10.1371/journal.pone.0320367)
Supplement: Attachment — Submitted filename: Responses to reviewers 2.docx [file pone.0320367.s006.docx]

**Responses to reviewers	11 Feb 2025**

Dear Editor,

We would like to thanks the reviewers and the subject editor for the thoughtful critiques of our manuscript. We have revised the methods and materials section of the manuscript based on the queries and hope that now you find it suitable for publication in the *PLOS ONE journal*. Our responses to the questions are detailed on the following page.

Masoumeh Fakhr Taha, PhD

Department of Stem Cells and Regenerative Medicine

Institute for Medical Biotechnology

National Institute of Genetic Engineering and Biotechnology (NIGEB)

Tehran, Iran

Emails: [mftaha@nigeb.ac.ir](mailto:mftaha@nigeb.ac.ir)

Phone: +98 912 [2999652](callto:+12999652)

Thank you for your response. For complete transparency, in your Methods, please clearly state 1) how the verbal consent for the use of adipose tissue samples for research purposes was documented and witnessed, and 2) state whether this verbal consent procedure was approved by the IRB.

- In response to the first question, as outlined in the methods and materials section, adipose tissue samples were collected from healthy women undergoing elective abdominoplasty, at Erfan-Niayesh Hospital, Tehran, Iran (from 2021.6.01 to 2022.12.30). While adipose tissue is typically considered as pathological waste destined for disposal, the collection of these samples was conducted in conjunction with the operating surgeon. Prior to the surgical procedure, non-written (verbal) consent was obtained from the patients, a process that was witnessed and documented by both the operating surgeon and the attending anesthesiologist. All donors agreed to the use of their adipose tissue samples for research purposes. Further information regarding the identities of the medical professionals involved in this documentation can be provided upon request.
- In regards to the second question, the documentations affirming the verbal consents were submitted to the Bioethics Committee of National Institute of Genetic Engineering and Biotechnology in order to obtain their approval. This committee conducted a thorough review of all study-related documents, including the consent forms, and subsequently approved the study, with the approval number: IR.NIGEB.EC.1400.2.26.C, dated: 2021.5.16.
