# Supplementary material for: Decellularized rat brain extracellular matrix effectively induces the dopaminergic differentiation of human adipose-derived stem cells
Source: PLoS One. 2025 Sep 2;20(9):e0320367. doi: 10.1371/journal.pone.0320367 (PMC12404474; doi:10.1371/journal.pone.0320367)
Supplement: Attachment — Submitted filename: Responces to Reviewers.docx [file pone.0320367.s007.docx]

Responses to reviewers	1 Jul 2025

Dear Editor,

We would like to thanks the reviewers and the subject editor for the thoughtful critiques of our manuscript. We have revised the manuscript and hope that now you find it suitable for publication in the PLOS ONE journal. Our point-by-point responses to the comments are detailed on the following pages.

Masoumeh Fakhr Taha, PhD

Department of Stem Cells and Regenerative Medicine

Institute for Medical Biotechnology

National Institute of Genetic Engineering and Biotechnology (NIGEB)

Tehran, Iran

Emails: taha_tmu73@yahoo.com, mftaha@nigeb.ac.ir

Phone: +98 912 2999652

Reviewer #3:

This study assesses the effect that decellularized rat brain slices have on the dopaminergic differentiation of human adipose-derived stem cells. However, there are some serious flaws in the study design which preclude conclusions being drawn.

Firstly, a preference towards dopaminergic differentiation cannot be claimed as other markers were not assessed by PCR or ICC (e.g. other neuronal types and/or glia). To claim a preference for dopaminergic differentiation, this should be compared to the subpopulations of other cell types.

***Response:*** Thank you for your valuable comment. We acknowledge that a definitive claim of dopaminergic lineage preference requires comparative analysis with other neuronal and glial subtypes. However, in this study, we focused on dopaminergic differentiation of hADSCs and did not examine other types of neurons or glial cells, for the following reasons:

1. The combination of SHH, FGF8, bFGF, and BDNF is a known cocktail for dopaminergic differentiation of stem cells. Sonic hedgehog (SHH) and fibroblast growth factor (FGF8) are traditional factors that are used in dopaminergic differentiation [1]. A combination of SHH and FGF8 can induce dopaminergic neurons at ectopic locations in the rat embryo, while SHH alone can only induce dopaminergic neurons at the dorsal-ventral axis [2]. In previous studies, a combination of SHH, FGF8, and bFGF has been used for dopaminergic differentiation of mouse and human embryonic stem cells [3, 4]. In 2007, Trzaska and colleagues [5] used a combination of SHH, FGF8, and bFGF for dopaminergic differentiation of human bone marrow-derived mesenchymal stem cells. Also, in 2011, Trzaska et al. generated DA-producing cells from adult human bone marrow-derived mesenchymal stem cells using SHH, FGF8, and bFGF and showed that electrophysiological functional DA neurons could be achieved by further treatment with BDNF [6]. The same combination of growth factors was used later by Shall et al. [7] and Phonchai et al. [8] for dopaminergic differentiation of rat bone marrow-derived mesenchymal stem cells and human amniotic fluid mesenchymal stem cells, respectively. In 2018, our team used this cocktail (SHH, FGF8, bFGF, and BDNF) for dopaminergic differentiation of human ADSCs and generated dopamine-secreting cells, with the same method used in the current study [9].
2. We would like to clarify that in the study by Trzaska et al. (2007), who differentiated BM-derived MSCs using the same medium as we employed, there was no detection of glial markers, such as GFAP and Olig2, in the differentiated cells. This absence of glial marker expression supports the notion that the differentiation was not directed toward glial lineages. The findings from Trzaska et al. further support the dopaminergic tendency under similar induction conditions. Nevertheless, we acknowledge the importance of including broader lineage markers in future studies to confirm lineage specificity.

Secondly, regarding the ICC data, this is the most critical data to claim dopaminergic differentiation but the data is problematic. Firstly both TH and Tuj1 are cytoplasmic markers. However, the staining here does not have any neuronal morphology, looks distinctly nuclear, and there is almost complete overlap with DAPI, especially the TH staining. This brings into question the validity of this data. Additionally, the authors state that a "significant portion" of the cells expressed TH and TUJ1. However, not only is this not statistically analysed, it is not even quantified.

***Response:*** Thank you for your critical observation regarding ICC images. We acknowledge the concerns raised about the staining pattern. However, it is important to note that for this experiments, the cells were cultured and differentiated on brain-derived scaffolds, which were subsequently fixed in formalin, processed into paraffin blocks, and sectioned for immunostaining. This process, while necessary for maintaining scaffold structure and enabling sectioning, may result in partial cell shrinkage or distortion of cytoplasmic structures, which could affect the apparent morphology and distribution of cytoplasmic markers, such as TUJ1 and TH proteins. This may partly explain the limited neuronal morphology and nuclear-like signal observed in the current images. Confocal microscopy is the preferred technique in this context; however, it exceeded the scope of our project and available resources.

Unfortunately, due to unforeseen circumstances beyond our control, we could not repeat the immunocytochemistry experiments. However, we have adjusted the contrast and brightness of the images in the revised figure to address the visualization concerns to some extent. Although we were unable to perform quantitative analysis of TH and TUJ1 expression, the results from gene expression (qPCR) and protein expression (western blot) collectively support the neuronal and dopaminergic differentiation of the ADSCs. Finally, it is worth noting that in our earlier research, using the same induction medium, we assessed the functional maturation of dopaminergic neurons generated from hADSC in 2D culture on gelatin-coated plates and validated through HPLC analysis that a considerable amount of dopamine was released in response to KCl-induced depolarization [10].

Thirdly, the control for the decellularized rat brain slices is not appropriate, or at least, other more appropriate controls should have been included. Why gelatin for example - this is not used in standard cell culture differentiation protocols and is not an ECM component highly expressed in brain tissue.

***Response:*** We agree with the reviewer that gelatin is not an ECM component highly expressed in the brain tissue. The primary objective of our study was not to compare our decellularized brain ECM with other 3-dimentional systems or various decellularized tissue ECMs. Instead, we aimed to develop a robust protocol for the decellularization of brain tissue. In our preliminary investigation, we examined multiple concentrations and treatment durations for sodium deoxycholate, Triton X-100, and trypsin/EDTA, alongside varying shaker incubator speeds. We found that the methodology outlined in this paper effectively removes the cells and genetic materials while retaining the ultrastructure and composition of the ECM throughout the decellularization process. Furthermore, we aimed to assess the dopaminergic differentiation of ADSCs on the brain ECM, as a 3-dimentional system, in comparison to our previously established two-dimensional system. Gelatin was selected as a coating material solely to enhance cell adhesion, facilitate cell spreading, and support cell proliferation and viability. It is noteworthy that gelatin-coated substrates are extensively used in cell culture applications for fibroblasts, neurons, and stem cells.

Finally, the data cannot be compared to existing literature as the time point for differentiation was not specified.

***Response:*** Thank you for your helpful comment. We acknowledge the omission of the total differentiation period in the original manuscript. This has now been incorporated in the revised version, where we have specified that the differentiation was performed over 12 days. We appreciate your attention to this detail.

Reviewer #4:

This is a general good original research. The authors must address these questions:

1. what are the main novelties of this research? It seems that a new method for decellularization has been conducted, but its efficacy and effectiveness has not been compared with common traditional approaches.

***Response:*** The main novelty of this research was firstly to establish a decellularization method for brain tissue that efficiently removes the cells and genetic material while retaining the ultrastructure and composition of the ECM during the decellularization process. Our goal in this project was not to compare our method with previously published ones, although it would have been better to do so.

Secondly, we aimed to assess the dopaminergic differentiation of human ADSCs on this decellularized brain ECM. As mentioned in the introduction “Previous studies have demonstrated the importance of using decellularized brain ECM to improve differentiation and maturation of neurons, with various cell sources such as neuroblastoma cells [11], induced pluripotent stem cell (iPSC)-derived neurons [12], neural stem cells [13, 14], and bone marrow-derived mesenchymal stem cells (BM-MSCs) [15] being utilized as the initial cells. However, no study has specifically examined the influence of brain ECM on the dopaminergic differentiation of stem cells in vitro”.

2- Since the control group of cells has received dopaminergic culture medium in two dimensional flask condition, the test group was cultured on 3D bio-scaffold. As that are substantial differences between two and three dimensional culture condition, the elevation of gene expression could not be exclusively related to the de-cellularized bio-scaffolds. More intricate and complex controlled condition is necessary to draw the claimed conclusions.

***Response:*** As mentioned in the answer to the previous question, in this project, we aimed to establish an efficient decellularization method for brain tissue and to examine dopaminergic differentiation of human ADSCs on it. Since we previously set up dopaminergic differentiation of human ADSCs in 2-dimensional culture in gelatin-coated plates, we used this system just to show by gene and protein analysis that dopaminergic differentiation on decellularized brain ECM is efficient. Albeit we agree with the reviewer that in the next step, we should compare our decellularization and differentiation methods with a previously established 3D culture system.

3- did the authors conduct any essay for cell sorting? Extraction of human adipose derived mesenchymal stem cells usually leads to a heterogeneous colony of the cells and scientific sorting approaches is necessary for compromising a homogeneous colony. Otherwise the results seem not to be repetitive.

***Response:*** As shown previously, ADSCs do not consistently express all characteristic MSC markers immediately after isolation, but some specific surface markers (e.g., CD105, CD166) increase during culture, while the expression of others decreases (e.g., CD34). During culture, the heterogeneity of ADSCs decreases, and it seems that the characteristic marker expression of ADSCs depends on the culture conditions and time in culture. ASCs in passages 2 or 3 are morphologically a *homogeneous population* of fibroblastoid cells. These cells uniformly express the characteristic markers of MSCs, including CD29, CD44, CD73, CD90, CD105, and CD166, and lack the expression of CD11b, CD14, CD31, and HLA-DR [16].

In our study, we assessed the third-passaged ADSCs for the expression of MSC markers by flow cytometry (Fig 1) and showed that more than 98% of the cells were positively stained with the antibodies against CD105, CD73 and CD90 proteins, while Only 0.3% of the cells showed expression of CD45 as a hematopoietic marker.

4- For further and more complete investigation it is needed to assess the expression of neuron exclusive genes in both mRNA and protein levels.

***Response:*** Thank you for the valuable comment. We examined the expression of neuron-specific genes, NSE and NEFL, at the mRNA level, and the results have been added to Figure 5. Additionally, neuron-specific proteins, TUJ1 and NEFL, have been previously assessed at the protein level, and their results are shown in Figure 7.

**Reviewer 5#**

Recommendation: Minor Revision. The manuscript generally meets PLOS ONE’s publication criteria in terms of scientific rigor, completeness of data, and ethical standards. The study is well designed and yields interesting results about using a decellularized rat brain extracellular matrix (ECM) to enhance dopaminergic differentiation of human adipose-derived stem cells (hADSCs). However, a few minor revisions are needed to improve clarity, consistency, and completeness. Below, I provide detailed comments on specific aspects:

**Scientific Validity and Methodological Rigor**

The experimental design is solid and appropriate for the research question. The authors compare hADSC differentiation on 3D decellularized brain scaffolds versus a standard 2D culture, which is a clear and logical way to test the influence of the ECM microenvironment. Strengths of the methodology include a thorough decellularization protocol (with chemical, enzymatic, and physical steps) and comprehensive validation of decellularization success (DAPI nuclear staining, H&E and Masson’s trichrome histology, DNA quantification, laminin immunostaining, and SEM imaging). These confirm that cellular material was effectively removed while preserving ECM structure – lending credibility to the scaffold’s integrity. The hADSCs are well-characterized prior to use (positive for MSC markers CD105, CD73, CD90 and able to undergo adipogenic/osteogenic differentiation), ensuring the starting cell population is appropriate and multipotent. The differentiation protocol is detailed and uses known dopaminergic-inductive factors (Neurobasal medium with SHH, FGF8, bFGF, later adding BDNF), which is scientifically sound. Multiple outcome measures are employed – including RT-PCR/qPCR for gene expression, immunocytochemistry/immunohistochemistry for neuronal markers, and Western blotting for protein levels – providing convergent evidence to support the conclusions. Overall, the study’s methods and analyses are rigorous and support the validity of the findings.

Areas for improvement: A few clarifications could further strengthen the rigor.

1. the authors should quantify some of the qualitative observations. For example, immunostaining revealed a “significant proportion” of cells positive for TH and TUJ1 in both conditions – it would improve the paper to quantify the percentage of cells expressing these markers on the scaffold vs. on 2D culture. This would give the reader a clearer idea of differentiation efficiency and how much the 3D ECM improves outcomes (e.g. “X% of cells were TH-positive on ECM vs. Y% on 2D”). If such quantification was done, it should be reported; if not, even a rough estimate or qualitative statement in the Discussion noting the difference in positive cell abundance would be helpful.

***Response:*** Thank you for your valuable feedback. We agree that quantifying the percentage of cells expressing dopaminergic-specific markers would strengthen the manuscript and provide readers a clearer idea of differentiation efficiency. However, the potential infiltration of certain cells into the scaffold, along with the fragility of neurons, presents challenges in isolating the cells from the scaffold and accurately quantifying their percentage. As a result, we were unable to provide exact percentages for TH-positive cells on the scaffold compared to those in 2D culture. It is worth noting that previous studies documenting neural differentiation on decellularized scaffolds also did not specify such percentages. Nevertheless, in our previously published research utilizing the same induction medium, we demonstrated through flow cytometry analysis that approximately 60% of hADSCs expressed TH following differentiation in 2D culture.

We hope this explanation clarifies our limitations and the context of our findings. We will consider including a brief statement in the discussion to offer a rough indication of differentiation efficiency within our system.

2. While the upregulation of dopaminergic genes (e.g. TH, GLI1, VMAT2, GIRK2) and exclusive expression of DDC and CALB in the 3D condition strongly support the conclusion, the manuscript could discuss the functional maturity of these differentiated cells. The authors are not expected to perform new experiments, but a brief acknowledgment that functional confirmation (such as dopamine release assays or electrophysiological properties) was beyond this study’s scope would give a balanced perspective. This could be framed as a future direction, reinforcing that the current evidence of marker expression is sufficient to call them “dopaminergic-like” cells, while true functional neurons would require additional verification.

***Response:*** We appreciate the reviewer's insightful comment. Due to the scope and resources of this study, we were unable to perform functional assays such as dopamine release measurements or electrophysiological recordings. In our earlier research, we assessed the functional maturation of dopaminergic neurons generated from hADSC in 2D culture on gelatin-coated plates and validated through HPLC analysis that a considerable amount of dopamine was released in response to KCl-induced depolarization [10]. In the present study, we did not examine the dopamine secretion by the differentiated cells on the brain ECM; however, based on our previous findings, we anticipate that the cells will secrete significant quantities of dopamine. We acknowledge that while our marker analysis strongly suggests a dopaminergic-like phenotype, comprehensive functional validation remains an important future direction to confirm neuronal maturity.

We will consider including a brief statement in the discussion to give the readers a clearer idea in this regard.

3. the inclusion of a control group where hADSCs are cultured on the scaffold without dopaminergic factors (to test if the ECM alone induces any neural differentiation) was not described. If such a control was not done, consider mentioning it as a potential future experiment to isolate the scaffold’s inherent inductive effect. These are minor suggestions to discuss or clarify; they do not detract from the core findings, which remain scientifically sound.

***Response:*** Unfortunately, we did not explore the differentiation of hADSCs on the brain scaffold in the absence of dopaminergic factors to determine whether the ECM alone can induce any neural differentiation. however, this topic can be addressed as a significant direction for future studies. We will consider incorporating a concise statement in the discussion to provide readers with a clearer understanding in this context.

**Reproducibility and Data Transparency**

The manuscript provides enough methodological detail for reproducibility. The decellularization procedure is outlined step-by-step with concentrations, durations, and equipment, which would allow other researchers to replicate the scaffold preparation. The source and preparation of hADSCs and the differentiation protocol are also described with sufficient detail (including media composition and growth factor concentrations). The use of multiple biological sources — five donors for adipose tissue and five rats for brain scaffolds — is commendable, as it increases confidence that the results are not from a single sample bias. It would be helpful to explicitly state how many independent experiments or biological replicates were analyzed for key results (e.g. were cells from all 5 donors used in parallel differentiations on separate scaffolds? Or were some analyses done in triplicate per condition?). Clarifying the number of biological replicates for the gene expression and protein analysis will underline the robustness of the data. Overall, the data presented (PCR gels, qPCR relative expression, immunostaining images, and Western blots) appear comprehensive and support the conclusions drawn.

***Response:*** Thank you for your insightful comment. Rats were sacrificed at three time points throughout the study, with one or two animals sacrificed each time. Following decellularization, differentiation was attempted on 3 to 4 separate brain sections from each rat. Therefore, for various analyses, different scaffolds and ASDC samples were used. This approach ensured that each analysis was performed on independent biological replicates from multiple scaffolds and ADSC sources, which underlines the robustness of the data. Regarding gene and protein expression, it should be mentioned that there were four replicates included for the gene expression and protein expression analyses.

Areas for improvement: In line with PLOS ONE’s data transparency policies,

1. The authors should ensure that all underlying data are made available. If not already done, include a Data Availability Statement indicating where numerical data (e.g. the quantitative values behind the qPCR bar graphs or Western blot densitometry) can be accessed. Additionally, when reporting statistical results, it’s good practice to indicate the n and what it represents. For instance, if “four replicates” were used in qPCR, clarify if these are four technical replicates or four independent differentiations (possibly from multiple donors).

***Response:*** All underlying data are available

2. Minor statistical considerations: since multiple genes were compared between two conditions, the authors might note whether any correction for multiple comparisons was applied or if each gene was treated as a separate planned comparison (the use of t tests with Welch’s correction is noted, which is appropriate for two-group comparisons assuming independent tests per gene/protein). These clarifications will further demonstrate the study’s reproducibility and adherence to transparency standards.

**Response:** As the comparisons were made between each treatment group and its respective control group, there was no need for any special correction.

**Ethical Compliance and Documentation**

The manuscript demonstrates strong ethical compliance. The authors clearly describe obtaining human adipose tissue with informed consent and approval, as well as the ethical oversight for animal use. Specifically, adipose samples were taken from patients undergoing elective surgery, with verbal informed consent documented by medical staff; this procedure was reviewed and approved by the institutional Bioethics Committee (approval ID provided: IR.NIGEB.EC.1400.2.26.C, dated 2021-05-16). The study states it was conducted in accordance with the Declaration of Helsinki, indicating attention to ethical standards for human tissue use. On the animal side, five Wistar rats were used for brain tissue, with housing and care as per standard conditions and all surgeries performed under deep anesthesia to minimize suffering. The animal protocol was approved by the same Bioethics Committee (approval ID: IR.NIGEB.EC.1400.2.26.B). The inclusion of approval numbers and dates for both human and animal procedures, as well as mention of adherence to relevant guidelines (Helsinki declaration for humans, laboratory animal care guidelines for animals), is excellent and meets journal requirements.

Areas for improvement: Only minor clarifications are needed.

1. It would be helpful to state the sex of the rats used, if known, as reporting sex is encouraged for animal studies (e.g., “five male Wistar rats” or “five female Wistar rats”). Currently only the strain and weight are given.

***Response:*** Five male Wistar rats were used in the present study. The sex of the rats was stated in the methods and materials section.

**Clarity of Writing and Presentation**

In general, the manuscript is well-written and structured. The Introduction provides a clear rationale, explaining the importance of ECM in neural development and the gap in knowledge regarding brain ECM’s effect on dopaminergic differentiation. The reader is given sufficient background to understand why this study matters (with citations to recent decellularization advances and prior uses of tissue-specific ECM). The Materials and Methods are detailed and logically ordered, and the Results flow in a coherent sequence (from stem cell characterization, to scaffold preparation/validation, to differentiation outcomes). The figures and tables cover all key data; for example, Figure 1 characterizes the ADSCs, Figures 2–4 likely show scaffold validation, Figure 5 covers gene expression, Figure 6 immunostaining, and Figure 7 Western blot results. This organization is easy to follow.

**Areas for improvement:**

A careful proofread is needed to correct some minor typographical and grammatical errors. For instance, in the Figure 6 description, “againest” should be corrected to “against.” In the Western blot methods, “ACBT served as the internal reference” appears to be a typo and should likely read “ACTB (β-actin) served as the internal reference protein.” Consistency in terminology should be checked: the text sometimes refers to “LMXA1” (in the Results and figure labels) whereas the correct gene/protein name is LMX1A – using one consistent spelling will avoid confusion. Similarly, “VMAT” is used in the text while the full name “VMAT2” is listed in the primers table; since VMAT2 is the specific vesicular monoamine transporter isoform measured, it may be clearer to use “VMAT2” throughout (or state at first use that “VMAT2 is referred to as VMAT”). Overall, these are minor edits that will enhance clarity.

***Response:*** Thank you for your insightful comment. We edited the manuscript carefully.

The authors should also ensure all acronyms are defined at first mention (most are, such as ECM, hADSC, etc., but double-check in case any slipped through).

***Response:*** We checked the manuscript carefully. Now, all acronyms are defined at first mention.

The figure captions could be slightly expanded for self-contained clarity – for example, when mentioning “negative control” in immunostaining images, specify that it refers to omission of primary antibody.

***Response:*** It is corrected now.

Finally, one suggestion for the Discussion: consider explicitly stating the main take-home message in a clear concluding sentence. The Conclusion section does summarize the findings and their significance for future applications, which is good. With these small revisions and proofreading corrections, the writing will be of high quality and easy for readers to understand.

***Response:*** We appreciate the reviewer's insightful comment. We edited the final paragraph of the discussion as follows:

“Collectively, these reports highlight the necessity of refining the methods of brain tissue decellularization and utilizing the decellularized bioscaffolds for the healing of central nervous system injuries, which formed the foundation of the present study. In summary, our findings demonstrated that an improved decellularization protocol involving two consecutive cycles yields a structurally preserved brain ECM that significantly promotes the dopaminergic differentiation of hADSCs, underscoring its potential for future application in brain tissue regeneration and healing of Parkinson’s disease.”

**References**

1.	Li, W., S. Chen, and J.Y. Li, *Human induced pluripotent stem cells in Parkinson's disease: A novel cell source of cell therapy and disease modeling.* Prog Neurobiol, 2015. **134**: p. 161-77.

2.	Ye, W., et al., *FGF and Shh signals control dopaminergic and serotonergic cell fate in the anterior neural plate.* Cell, 1998. **93**(5): p. 755-66.

3.	Chung, S., et al., *The homeodomain transcription factor Pitx3 facilitates differentiation of mouse embryonic stem cells into AHD2-expressing dopaminergic neurons.* Mol Cell Neurosci, 2005. **28**(2): p. 241-52.

4.	Roy, N.S., et al., *Functional engraftment of human ES cell-derived dopaminergic neurons enriched by coculture with telomerase-immortalized midbrain astrocytes.* Nat Med, 2006. **12**(11): p. 1259-68.

5.	Trzaska, K.A., E.V. Kuzhikandathil, and P. Rameshwar, *Specification of a dopaminergic phenotype from adult human mesenchymal stem cells.* Stem Cells, 2007. **25**(11): p. 2797-808.

6.	Trzaska, K.A. and P. Rameshwar, *Dopaminergic neuronal differentiation protocol for human mesenchymal stem cells.* Methods Mol Biol, 2011. **698**: p. 295-303.

7.	Shall, G., et al., *Effects of Passage Number and Differentiation Protocol on the Generation of Dopaminergic Neurons from Rat Bone Marrow-Derived Mesenchymal Stem Cells.* Int J Mol Sci, 2018. **19**(3).

8.	Phonchai, R., et al., *Potential effects and molecular mechanisms of melatonin on the dopaminergic neuronal differentiation of human amniotic fluid mesenchymal stem cells.* Neurochem Int, 2019. **124**: p. 82-93.

9.	Soheilifar, M.H., et al., *Generation of Dopamine-Secreting Cells from Human Adipose Tissue-Derived Stem Cells In Vitro.* Rejuvenation Res, 2018. **21**(4): p. 360-368.

10.	Faghih, H., et al., *Directed differentiation of human adipose tissue-derived stem cells to dopaminergic neurons in low-serum and serum-free conditions.* Neurosci Lett, 2019. **708**: p. 134353.

11.	Medberry, C.J., et al., *Hydrogels derived from central nervous system extracellular matrix.* Biomaterials, 2013. **34**(4): p. 1033-1040.

12.	DeQuach, J.A., et al., *Decellularized porcine brain matrix for cell culture and tissue engineering scaffolds.* Tissue Engineering Part A, 2011. **17**(21-22): p. 2583-2592.

13.	Crapo, P.M., et al., *Effects of biologic scaffolds on human stem cells and implications for CNS tissue engineering.* Tissue Engineering Part A, 2013. **20**(1-2): p. 313-323.

14.	De Waele, J., et al., *3D culture of murine neural stem cells on decellularized mouse brain sections.* Biomaterials, 2015. **41**: p. 122-131.

15.	Zhang X, W.B., Hong H, Wang Y, Liu J, Liu C, Chen X, *Decellularized extracellular matrix and mesenchymal stem cells promote recovery in traumatic brain injury by synergistically enhancing neurogenesis and attenuating neuroinflammation.* Journal of Materials Science and Technology, 2023. **138**: p. 17-28.

16.	Baer, P.C., *Adipose-derived mesenchymal stromal/stem cells: An update on their phenotype in vivo and in vitro.* World J Stem Cells, 2014. **6**(3): p. 256-65.
